# Supplementary material for: Betaine and L-Carnitine Synergistically Influence the Metabolome and Immune Response in Dogs
Source: Animals (Basel). 2024 Jan 23;14(3):357. doi: 10.3390/ani14030357 (PMC10854714; doi:10.3390/ani14030357)
Supplement: Supplementary file 1 [file animals-14-00357-s001.zip › animals-2736176-supplementary.pptx]

## Slide 1
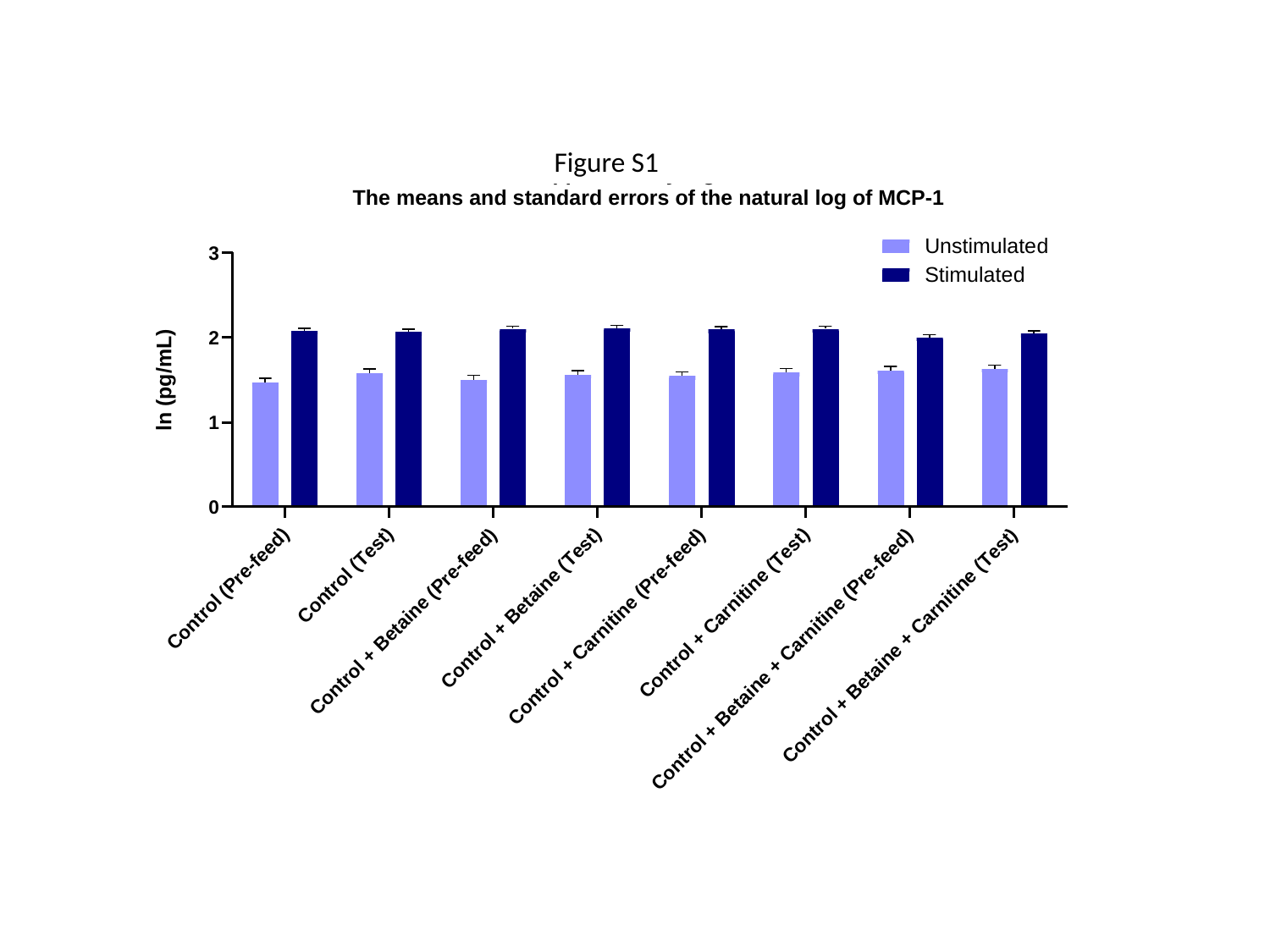

Figure S1

## Slide 2
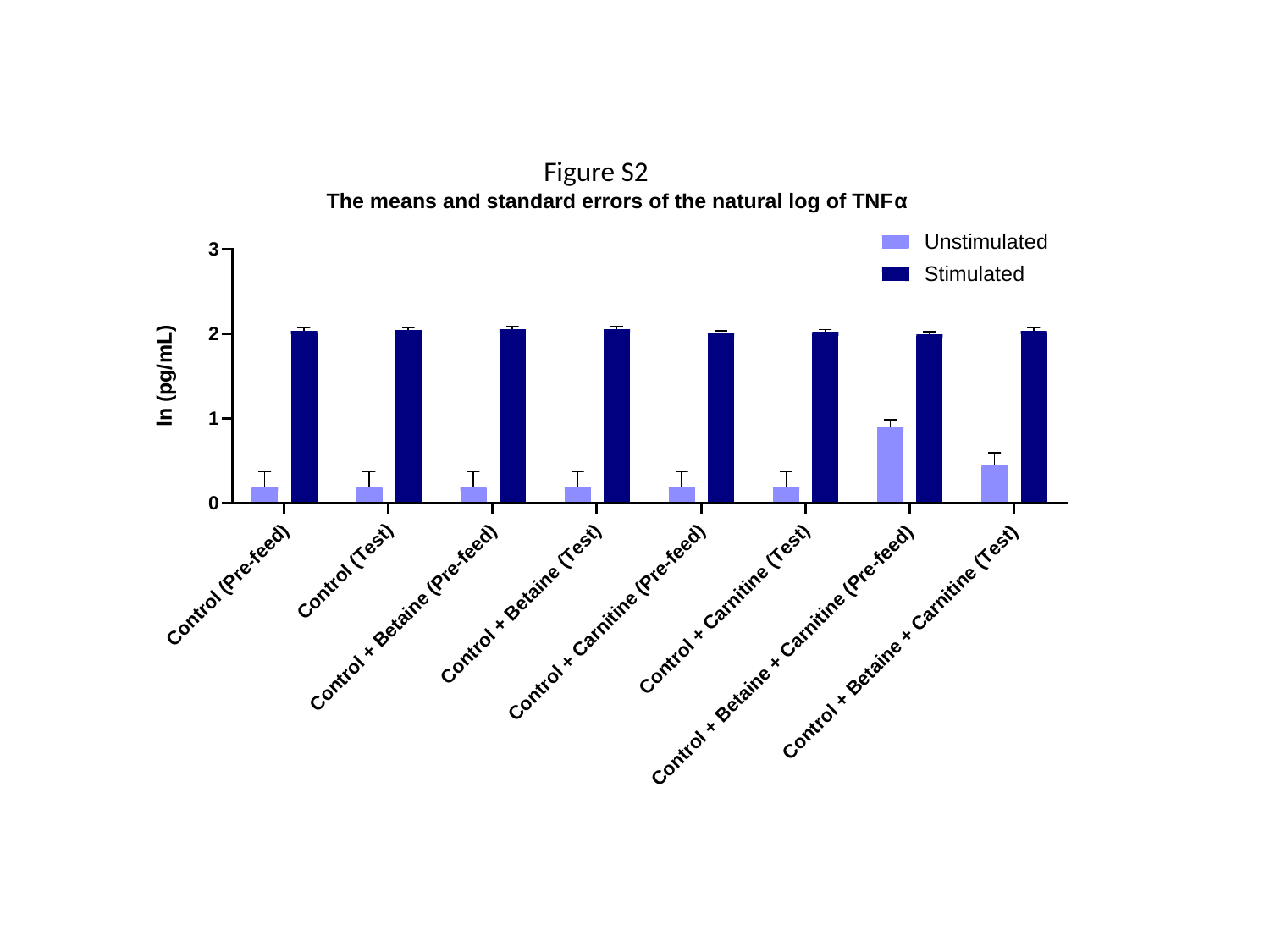

Figure S2

## Slide 3
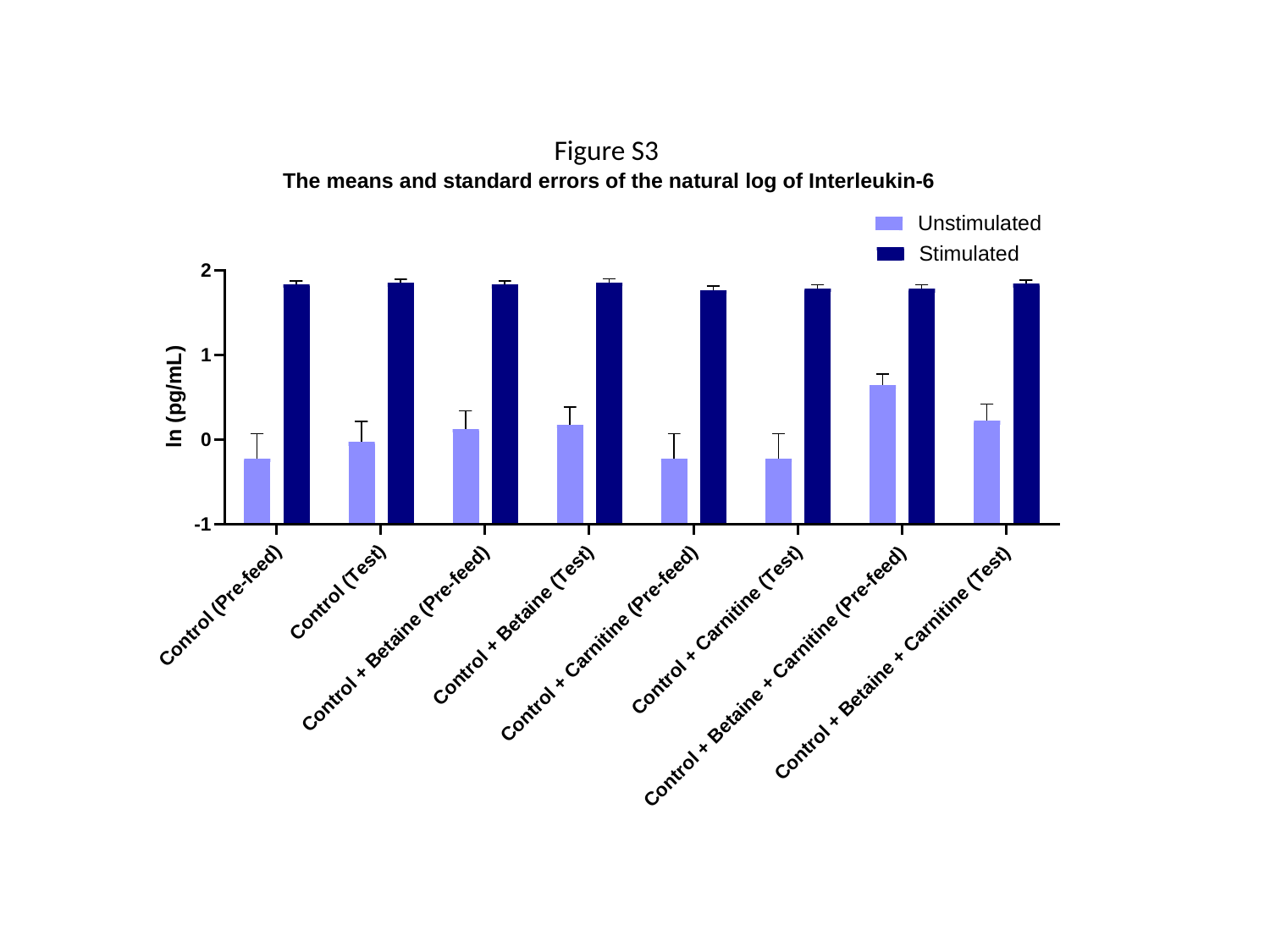

Figure S3

## Slide 4
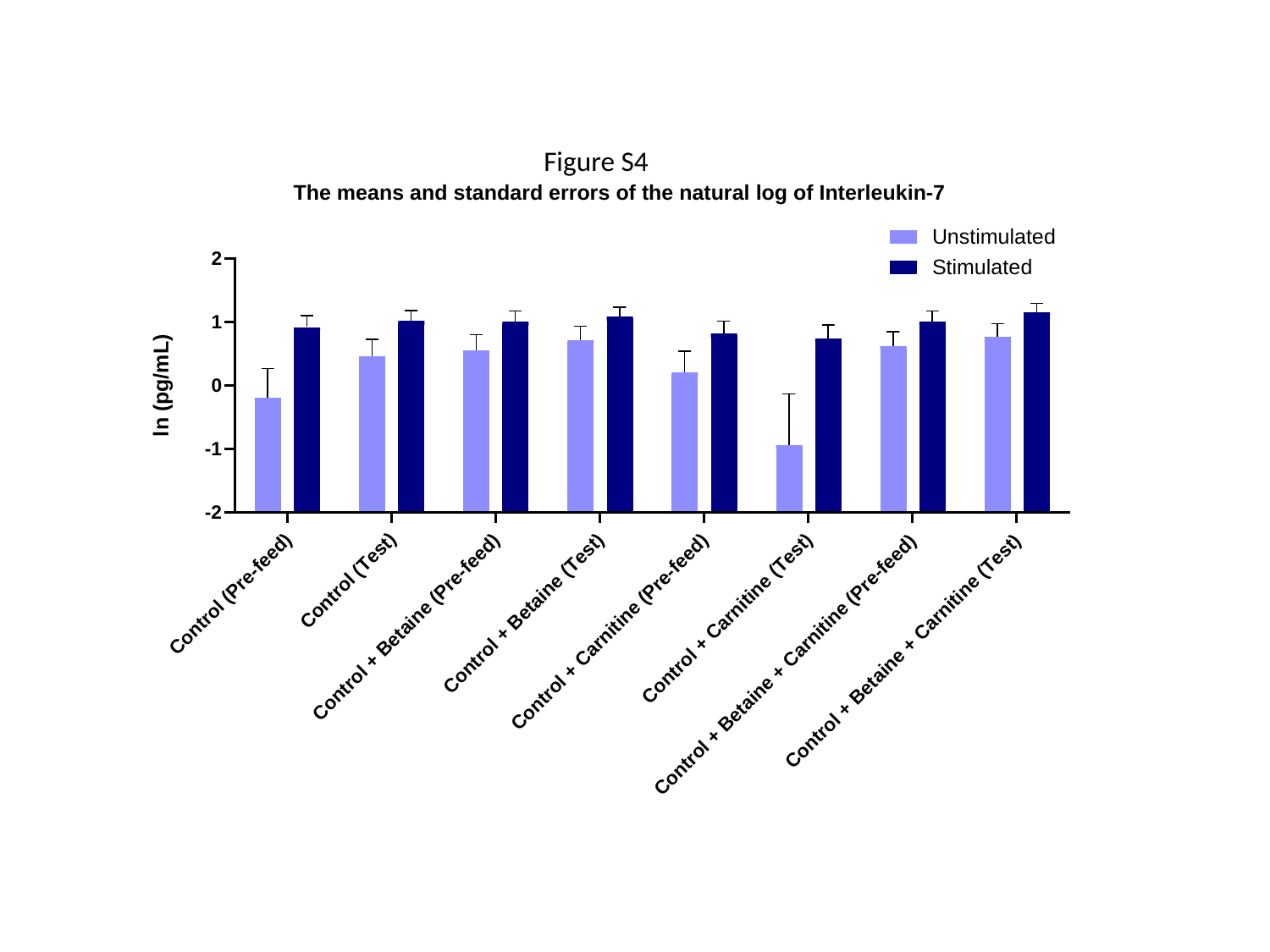

Figure S4

## Slide 5
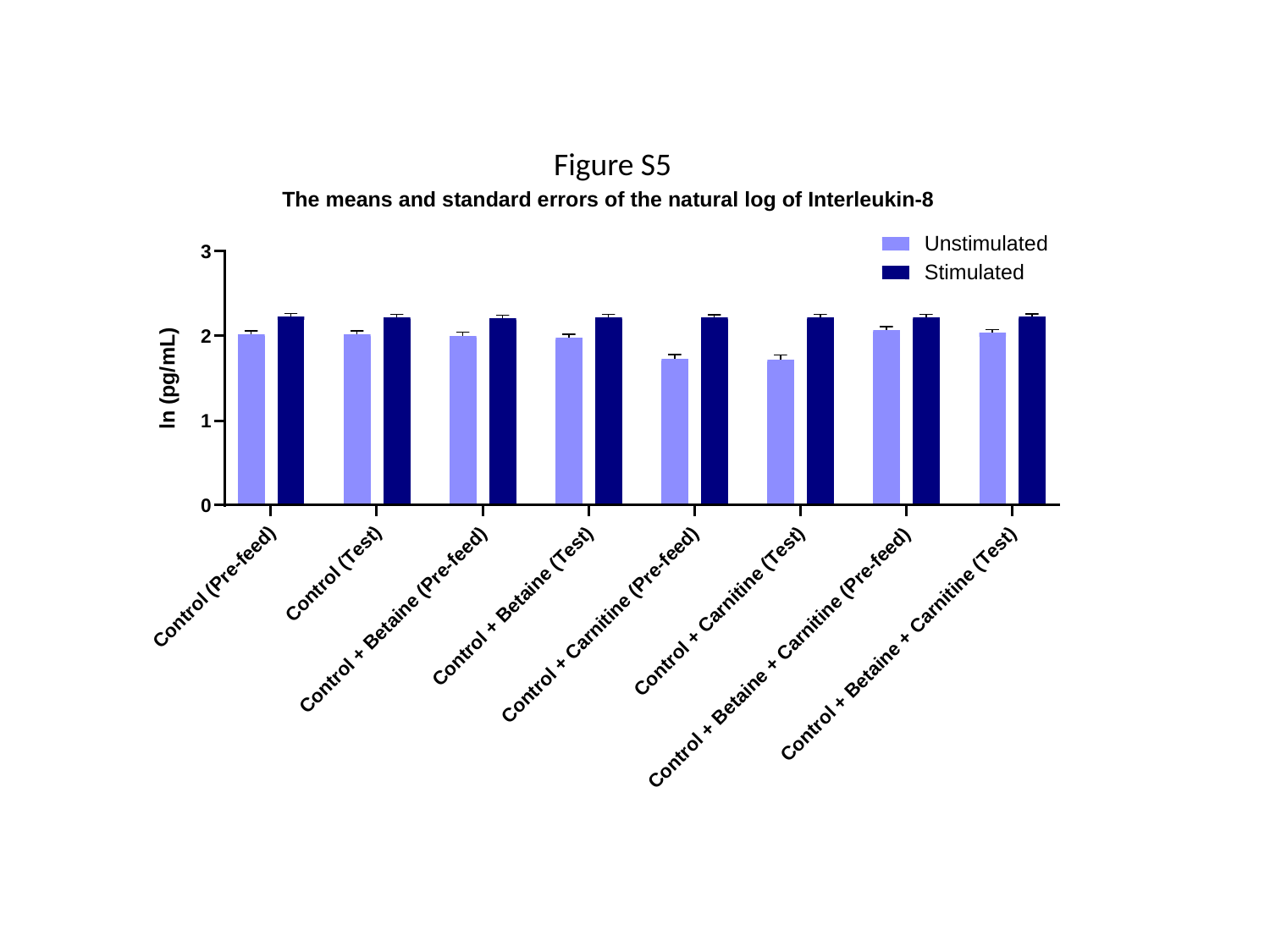

Figure S5

## Slide 6
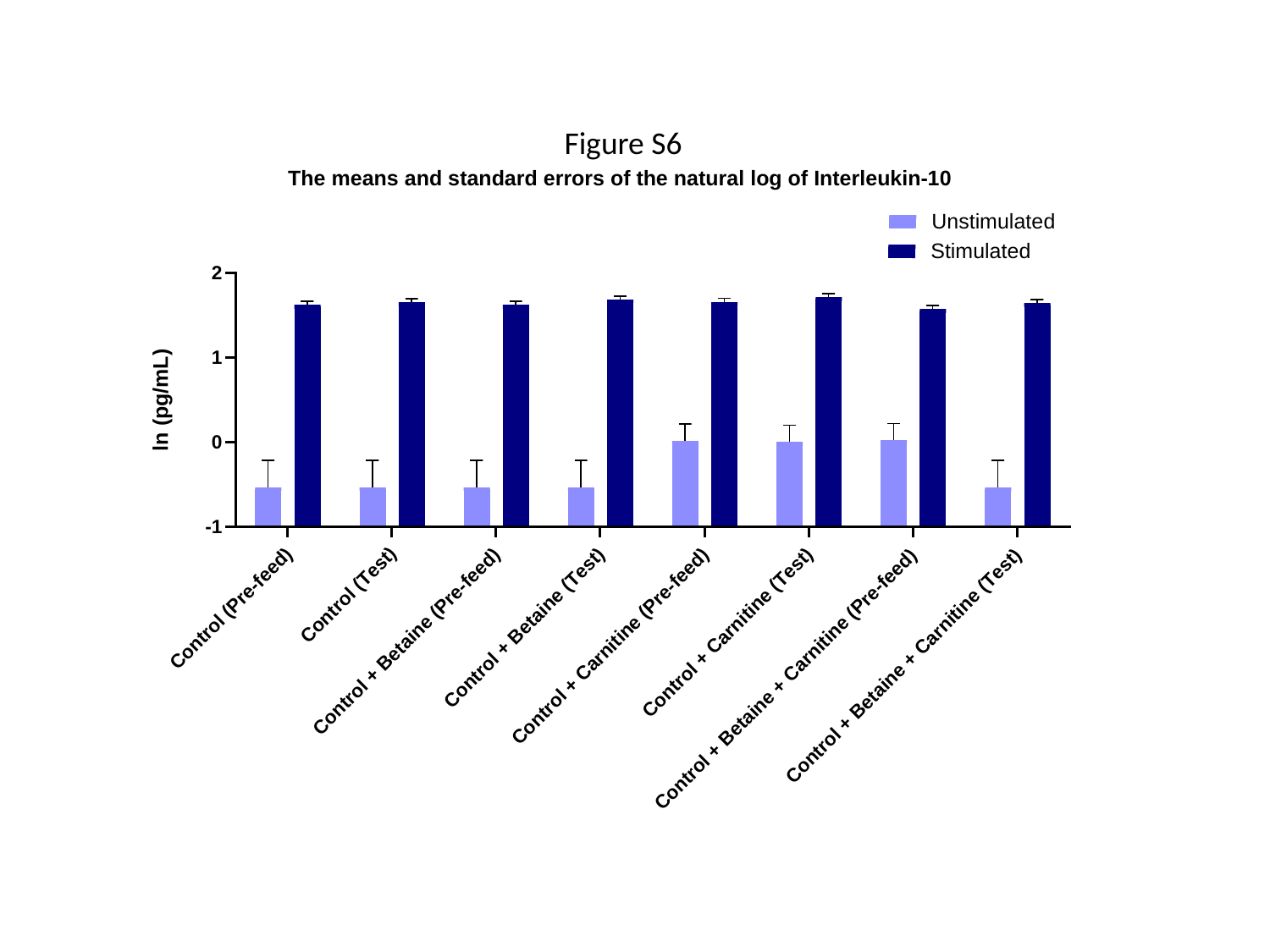

Figure S6

## Slide 7
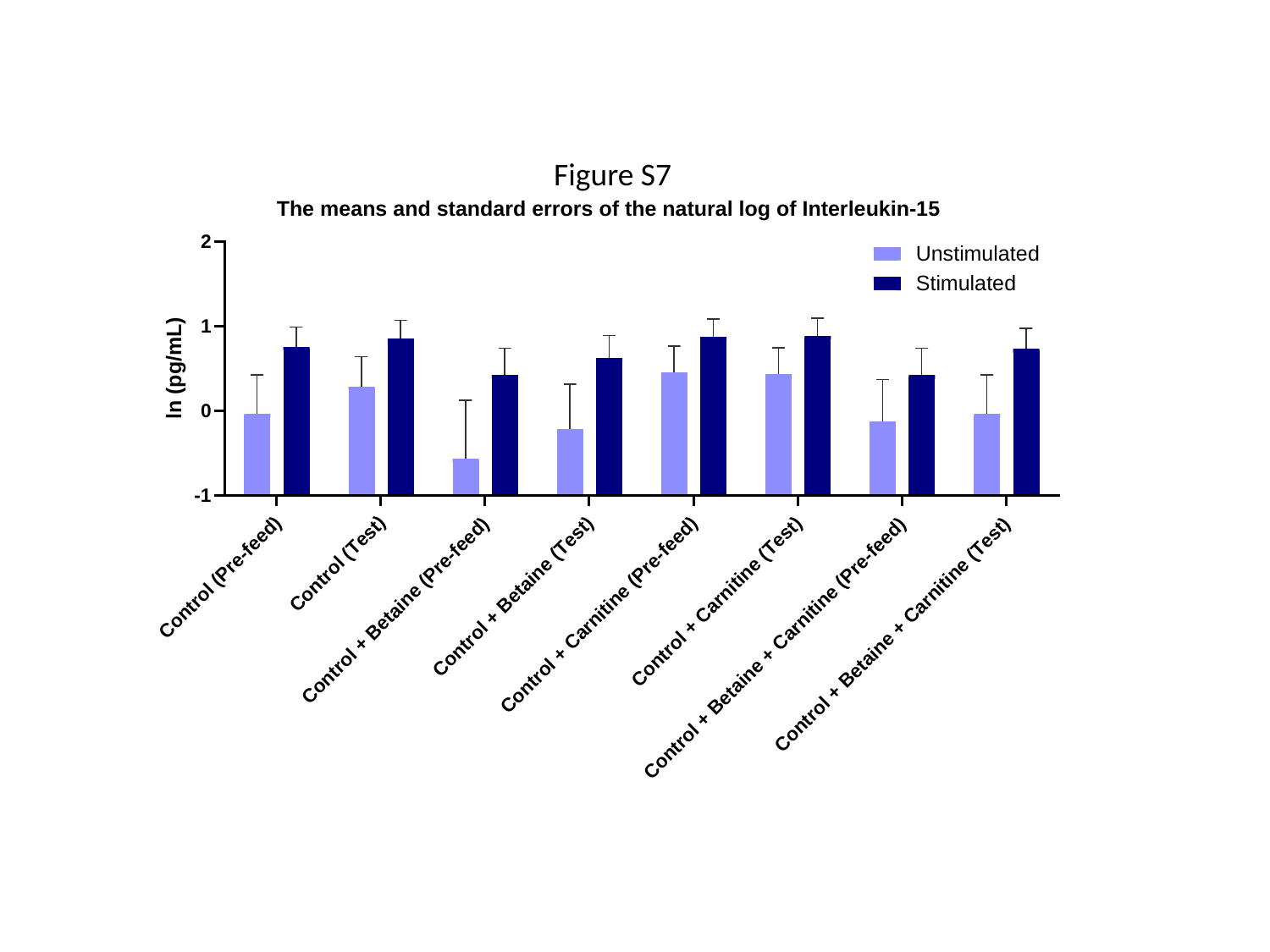

Figure S7
